# Hyperglycemia regulates thioredoxin-ROS activity through induction of thioredoxin-interacting protein (TXNIP) in metastatic breast cancer-derived cells MDA-MB-231

**DOI:** 10.1186/1471-2407-7-96

**Published:** 2007-06-07

**Authors:** Francesco Turturro, Ellen Friday, Tomas Welbourne

**Affiliations:** 1 Department of Medicine, Feist-Weiller Cancer Center, Louisiana State University Health Science Center, 1501 Kings Highway, Shreveport, Louisiana, 70103, USA; 2 Gene Therapy Program, Louisiana State University Health Science Center, 1501 Kings Highway, Shreveport, Louisiana, 70103, USA; 3 Department of Molecular and Cellular Physiology, Louisiana State University Health Science Center, 1501 Kings Highway, Shreveport, Louisiana, 70103, USA

## Abstract

**Background:**

We studied the RNA expression of the genes in response to glucose from 5 mM (condition of normoglycemia) to 20 mM (condition of hyperglycemia/diabetes) by microarray analysis in breast cancer derived cell line MDA-MB-231. We identified the thioredoxin-interacting protein (TXNIP), whose RNA level increased as a gene product particularly sensitive to the variation of the level of glucose in culture media. We investigated the kinesis of the TXNIP RNA and protein in response to glucose and the relationship between this protein and the related thioredoxin (TRX) in regulating the level of reactive oxygen species (ROS) in MDA-MB-231 cells.

**Methods:**

MDA-MB-231 cells were grown either in 5 or 20 mM glucose chronically prior to plating. For glucose shift (5/20), cells were plated in 5 mM glucose and shifted to 20 mM at time 0. Cells were analyzed with Affymetrix Human U133A microarray chip and gene expression profile was obtained. Semi-quantitative RT-PCR and Western blot was used to validate the expression of TXNIP RNA and protein in response to glucose, respectively. ROS were detected by CM-H2DCFDA (5–6-chloromethyl-2',7'-dichlorodihydrofluorescein diacetate) and measured for mean fluorescence intensity with flow cytometry. TRX activity was assayed by the insulin disulfide reducing assay.

**Results:**

We found that the regulation of TXNIP gene expression by glucose in MDA-MB-231 cells occurs rapidly within 6 h of its increased level (20 mM glucose) and persists through the duration of the conditions of hyperglycemia. The increased level of TXNIP RNA is followed by increased level of protein that is associated with increasing levels of ROS and reduced TRX activity. The inhibition of the glucose transporter GLUT1 by phloretin notably reduces TXNIP RNA level and the inhibition of the p38 MAP kinase activity by SB203580 reverses the effects of TXNIP on ROS-TRX activity.

**Conclusion:**

In this study we show that TXNIP is an oxidative stress responsive gene and its expression is exquisitely regulated by glucose level in highly metastatic MDA-MB-231 cells.

## Background

We have recently described the gene expression profile (GEP) of the highly metastatic breast cancer-derived MDA-MB-231 cells in response to hyperglycemia *in vitro *tissue culture [1]. Among all the RNAs that showed significant changes in response to increased level of glucose, the level of RNA of the thioredoxin-interacting protein (TXNIP), also known as vitamin D_3 _up-regulated protein-1 (VDUP-1) was one of the most representatives [1].

The emphasis on the role of both insulin and insulin-like growth factors 1 (IGF-1) or IGF binding proteins (IGFBPs), together with the insulin-mediated regulation of fat distribution, availability of sex hormone binding globulin (SHBG), and sex hormones in the regulation of proliferation and growth of breast derived cells, has undermined the relevance of glucose by itself in breast cancer [2-5]. In complex organisms such as vertebrates, it becomes very difficult to discriminate glucose effects on gene transcription from those related to either insulin or glucagon, whose secretions are regulated by glucose [6]. However, the use of established cell lines has allowed more recently the study of the direct effect of glucose on the proliferation of hepatocytes [6].

It has been recently shown that the promoter region of the TXNIP gene contains carbohydrate response elements (ChoRE) conferring the described glucose responsiveness in murine pancreatic β cells [7,8]. The function of TXNIP as a modulator of the redox system through binding of the thioredoxin (TRX) active cysteine residues has been elucidated in recent studies [9,10]. The intracellular redox balance is maintained by reactive oxygen species (ROS)-scavenger systems, mainly represented by the glutathione and the TRX systems [11]. A recent study has also shown that hyperglycemia causes oxidative stress through inhibition of TRX function by TXNIP in human aortic smooth muscle cells [12].

In the current study, we initially validated the results of the glucose-mediated expression of TXNIP obtained by the initial GEP screening by assessing the levels of mRNA by RT-PCR as compared to the level of TRX in breast cancer derived cell line MDA-MB-231 [1]. We then assessed the time course of the variation of both RNA and protein TXNIP levels in response to increased level of glucose, and finally demonstrate that TXNIP regulates ROS levels through TRX-activity in MDA-MB-231 cells.

## Methods

### Cell lines and tissue culture

Breast cancer-derived MDA-MB-231 cells were purchased from American Type Culture Collection (Mannassas, VA). Cells were grown to confluence in Dulbecco's modified Eagle's medium (DMEM) plus 10% fetal calf serum (FCS) containing 28 mM/L sodium bicarbonate, 10 mM/L sodium pyruvate, 5 mM/L D-glucose, and 2 mM/L L-glutamine at 37°C (pH 7.4). The cells were maintained in 5 or 20 mM/L D-glucose chronically prior to plating. For glucose shift, cells were plated in 5 mM/L D-glucose and shifted to 20 mM at time 0.

### Semi-quantitative RT-PCR

Total RNA was isolated using Aquapure RNA isolation kit (Bio-Rad, Hercules, CA) and first strand c-DNA synthesis by iScript c-DNA amplification kit (Bio-Rad) according to manufacture's protocol. Primers were designed with Beacon Designer program (Premier Biosoft, Palo Alto, CA) as it follows: TXNIP SENSE 5'-TCA Tgg TgA Tgt TCA AgA AgA TC-3'; ANTISENSE 5'-ACT TCA CAC CTC CAC TAT C-3'; TRX SENSE 5'-CAg ggg AAT gAA AgA AAg g-3'; ANTISENSE 5'-CAA ggT gAA gCA gAT Cg-3'; β-ACTIN SENSE 5'-TTT gAA TgA TgA gCC TTT gTg-3'; ANTISENSE 5'-TCA gTg TAC Agg TAA gCC CT-3'. PCR products for TXNIP, TRX and β-actin were amplified using PCR-Supermix (Promega, Madison, WI) using 1/10 of the cDNA reaction mix with the following profile for 30 cycles: denaturation 95°C for 1 min, annealing 50° for 1 min and extension 68° for 1 min. PCR products were run by electrophoresis on 3% agarose gel and stained with Syber-Sale DNA stain (Invitrogen, Carlsbad, CA). For semi-quantization amounts of RNA was estimated by the relative intensity against the relative intensity of β-actin.

### Inhibition of glucose transport

Cells (5 × 10^5^) were plated in 6 well-dishes in duplicate in 5 mM glucose and allowed to attach overnight. Cells receiving phloretin were pre-treated for 1 h prior to glucose shift. At time 0, cells were either maintained in 5 mM glucose or switched to 20 mM glucose + 300 μM phloretin. Cells were harvested after 6 h for RNA isolation as previously described.

### Western and immunoblot analysis

For analysis of chronic TXNIP protein expression cells were maintained indefinitely in DMEM with 5 or 20 mM glucose. For the time course of TXNIP protein level, cells maintained in 5 mM glucose were plated in 6 well-plates. At time 0, fresh medium containing 20 mM glucose was added and cells were harvested at indicated time-points. From cell lysates 100 μg of total proteins was run on 10% SDS PAGE gel by electrophoresis and blotted with rabbit polyclonal antibody to TXNIP (Invitrogen, Carlsbad, CA). Blots were stripped and reprobed for β-actin (Labvison, Fremont, CA) as loading control.

### ROS assay, TRX activity, and p38 MAP kinase inhibition

ROS was detected by CM-H2DCFDA (5- [and -6]-chloromethyl-2',7'-dichlorodihydrofluorescein diacetate, acetyl ester (Molecular Probes). Cells were loaded with 10 μM DCFDA for 30 min at 37°C, 5% CO_2 _in PBS. Cells were washed and returned to media for a 30 min recovery period. Mean fluorescence intensity was used as measure of ROS as determined by flow cytometry FACS Calibur using CellQuest Pro 5.2 on 1 × 10^4 ^cells (BD Bioscience, San Jose, CA) [13]. TRX activity was assessed by the insulin disulfide reducing assay as previously described [13]. Briefly, 50 μg of cell extract was incubated in reducing buffer (50 mM HEPES, 1 mM EDTA, 1 mg/ml BSA and 2 mM DTT) at 37°C for 15 min. After the addition of reaction buffer, the reaction was started by the addition of 5 μl bovine thioredoxin reductase (100 U/ml) or water as negative control followed by 20 min incubation. The reaction was stopped by the addition of 0.5 ml of stopping buffer (6 M guanidine-HCl, and 1 mM DNTB). The absorbance at 412 nm was measured. For p38 MAP kinase inhibition experiments, 5 × 10^5 ^cells were initially seeded and maintained in 5 or 20 mM glucose media, and then the cells at 20 mM glucose were treated for 24 h with 20 μM specific inhibitor SB203580 (Sigma, St Louis, MO).

### Statistical analysis

Experiments were carried in duplicates or triplicates as specified. Differences between treatments were evaluated by ANOVA or student's t-test. Differences were accepted as significant if p < 0.05 (two-tailed).

## Results

### Hyperglycemia regulates the levels of TXNIP RNA, but not of TRX RNA

Our GEP data were obtained from MDA-MB-231 cells chronically maintained in media containing 5 or 20 mM glucose prior to plating as previously described [1]. In order to assess the "subacute" glucose shift, cells were plated in 5 mM glucose and shifted to 20 mM at time 0 [1]. Cell viability and proliferation was assessed by trypan blue exclusion at various time-points and when a significant difference in growth (separation of the curves for each cell group 5, 5/20 and 20 mM glucose) was seen at 12 h (data not shown and presented in Ref. 1) RNA was harvested and hybridized to Affymetrix Human μ133A chips as described in the Methods. GeneSpring 7.2 was used to average results and perform analysis between treatment groups [1]. Final profiles were generated using minimum of 1.5 fold change function [1].

**Figure 1:**
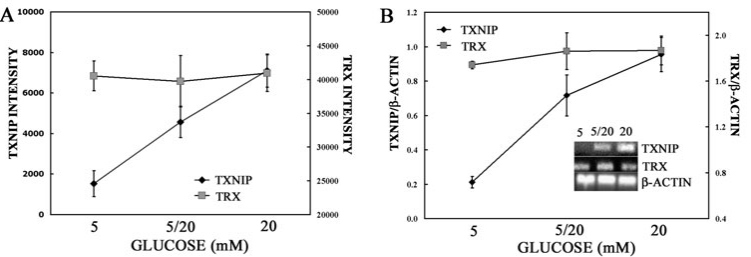
**TXNIP and TRX expression in response to glucose assessed by gene expression profile (GEP) and semi-quantitative PCR. **A) MDA-MB-231 cells were grown either in 5 or 20 mM glucose chronically prior to plating. For glucose shift (5/20), cells were plated in 5 mM glucose and shifted to 20 mM at time 0. Cells were harvested at 12 h based on previous growth curves obtained at specified glucose concentration and RNA was isolated, labeled and hybridized to Affymetrix Human U133A microarray chip. Average derived from duplictaes is shown as relative expression of TXNIP and TRX RNAs, respectively. B) TXNIP and TRX RNA message levels were detected by semi-quantitative PCR in MDA-MB-231 cells grown in the same conditions as in A. Average relative levels as compared to control β-actin RNA of TXNIP and TRX RNA messages from duplicates are represented. Representative gel electrophoresis of PCR products obtained from duplicate experiments is shown in the inset.

As shown in Figure 1A according to our GEP data, the level of TXNIP increased from 1528 ± 637 in MDA-MB-231 cells grown in culture media containing 5 mM glucose to 4572 ± 765 when the cells were placed in 20 mM glucose. Cells chronically grown in media containing 20 mM glucose showed the highest level at 7104 ± 823, while the TRX level remained unchanged with the increasing level of glucose concentration (Figure 1A). To validate the GEP data, we assessed the level of TXNIP and TRX message by semi-quantitative RT-PCR and reported as relative level to the control RNA β-actin. Relative levels of TXNIP increased with increasing levels of glucose (0.21 ± 0.03 at 5 mM vs 0.72 ± 0.12 at 5/20 mM vs 0.95 ± 0.10 at 20 mM; p < 0.05) resulting in an overall 4.3 fold increase from 5 mM to 20 mM (Figure 1B). On the contrary, TRX message remained unchanged at the various glucose levels (Figure 1B). These results validated the GEP data confirming that the level of TXNIP RNA significantly increased upon "subacutely" increasing and persistently maintaining elevated levels of glucose (chronic conditions), while TXR RNA message remained unchanged.

### Hyperglycemia acutely regulates the increase of TXNIP RNA level and is dependent on the cellular level of glucose

**Figure 2:**
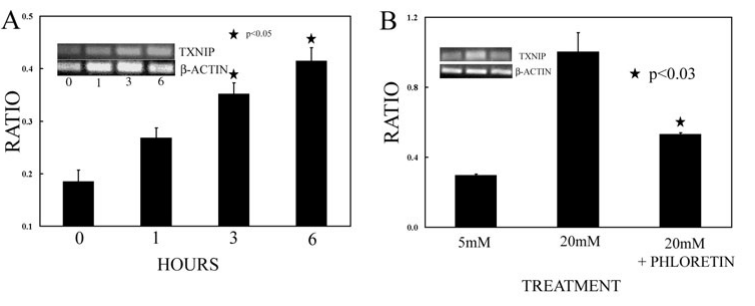
**Time course of the TXNIP RNA expression and response to inhibition of glucose transporter. **A) MDA-MB-231 cells were chronically grown at 5 mM and then switched to 20 mM glucose at t = 0. RNA was measured by semi-quantitative PCR at the indicated time points and shown as average ratio of control β-actin RNA levels derived from duplicates. Representative gel electrophoresis of PCR products obtained from 2 experiments is shown in the inset. B) Cells were grown chronically at 5 mM and then switched to 20 mM glucose at t = 0. RNA was measured by semi-quantitative PCR at 6 h and shown as average ratio of control β-actin RNA levels from duplicates. For inhibition of the glucose transporter study cells were pre-treated for 1 h with 300 μM phloretin. Representative gel electrophoresis of PCR products obtained from 2 experiments is shown in the inset.

The level of TXNIP RNA in the initial GEP and in the validating semi-quantitative RNA analysis were derived from conditions of "chronic" or stable growth in 20 mM glucose, and even in conditions of shifting from 5 to 20 mM glucose, the measurements occurred after 12 h from the shift (Figure 1). For the purpose of determining the earliest time after increasing the glucose concentration the rise of the TXNIP RNA level took place, we investigated the time course of RNA expression by semi-quantitative PCR analysis. As shown in Figure 2, the TXNIP/β actin level ratio significantly increased within 1 h and significantly (p < 0.05) continued to increase up to 6 h (0.14 ± 0.02 at 0 h; 0.22 ± 0.02 at 1 h; 0.30 ± 0.02 at 3 h; 0.36 ± 0.03 at 6 h). These data show that the effect of glucose on TXNIP RNA level is rapid and increases with exposure to high levels of glucose (20 mM) within hours.

To assess whether the glucose-induced increase of TXNIP RNA at 6 h was regulated by the intracellular level of glucose, we inhibited the transport of the glucose with phloretin which is an effective though not specific inhibitor of the GLUT1 transporter as previously shown [14,15]. The inhibitor significantly reduced the level of TXNIP RNA (5 mM vs 20 mM, p = 0.012; 20 mM vs 20 mM + phloretin, p = 0.027; 5 mM vs 20 mM + phloretin, p = 0.002) as shown in Figure 2B and expressed as ratio to control (0.30 ± 0.01 at 5 mM; 1.00 ± 0.11 at 20 mM; 0.53 ± 0.01 at 20 mM + phloretin).

### Hyperglycemia-mediated increase of TXNIP RNA correlates with TXNIP protein level

**Figure 3:**
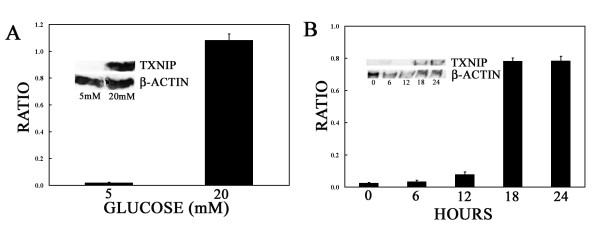
**TXNIP protein expression and time chase of the TXNIP protein expression in response to glucose. **A) MDA-MB-231 cells were grown either in 5 or 20 mM glucose chronically prior to plating as described. Cells were harvested and total proteins obtained from cell lysates were run on 10% SDS PAGE gel by electrophoresis and blotted with rabbit polyclonal antibody to TXNIP. Blots were stripped and reprobed for β-actin as loading control to estimate the average ratio of band intensity from duplicates as shown. Representative Western blot obtained from 2 experiments is shown in the inset. B) MDA-MB-231 cells were chronically grown at 5 mM and then switched to 20 mM glucose at t = 0 and treated as described. Cells were harvested at the time points indicated and total proteins obtained from cell lysates were run on 10% SDS PAGE gel by electrophoresis and blotted with rabbit polyclonal antibody to TXNIP. Blots were stripped and reprobed for β-actin as loading control to estimate the average ratio of band intensity from duplicates as shown. Representative Western blot obtained from 2 experiments at each time point is shown in the inset.

For the purpose of assessing whether the increased level of TXNIP RNA determined by prolonged conditions of hyperglycemia (20 mM, chronic conditions) were correlated with correspondent elevated levels of TXNIP protein, we analyzed the expression of the protein in MDA-MB-231 cells grown "chronically" and stably in 20 mM glucose tissue culture media by Western blot. As shown in Figure 3A, the intensity of the band (expressed as ratio to the reference β-actin protein) within the expected molecular weight (50 kDa) of the TXNIP protein which was absent at 5 mM notably increased in the cells grown at 20 mM glucose (0.02 ± 0.005 at 5 mM vs 1.08 ± 0.05 at 20 mM glucose). This finding showed that TXNIP protein level correlated with RNA message and glucose level in MDA-MB-231 cells.

In order to evaluate the earliest time of increasing levels of TXNIP protein caused by the increasing levels of glucose (acute conditions), we executed the time course/Western blot analysis within hours from the switch 5/20 mM using MDA-MB-231 cells chronically grown at 20 mM glucose as control. As shown in Figure 3B the intensity of the band (expressed as ratio to the reference β-actin protein) in the expected MW range of TXNIP protein increased over the time reaching a peak at 18 h (0.03 ± 0.001 at 0 h, 0.03 ± 0.01 at 6 h, 0.08 ± 0.02 at 12 h, 0.78 ± 0.09 at 18 h), which occurred 12 h after the peak level of RNA message (Figure 2A). The peak level of TXNIP protein persisted at 24 h (Figure 3B, ratio at 24 = 0.79 ± 0.03) confirming the fact that TXNIP protein level remains persistently elevated if the glucose level remains elevated at 20 mM as shown in chronic conditions. These data confirm that hyperglycemia affects acutely and chronically RNA and protein levels of TXNIP causing persistent elevated levels.

### TXNIP/ROS/TRX axis is inversely related in conditions of hyperglycemia

**Figure 4:**
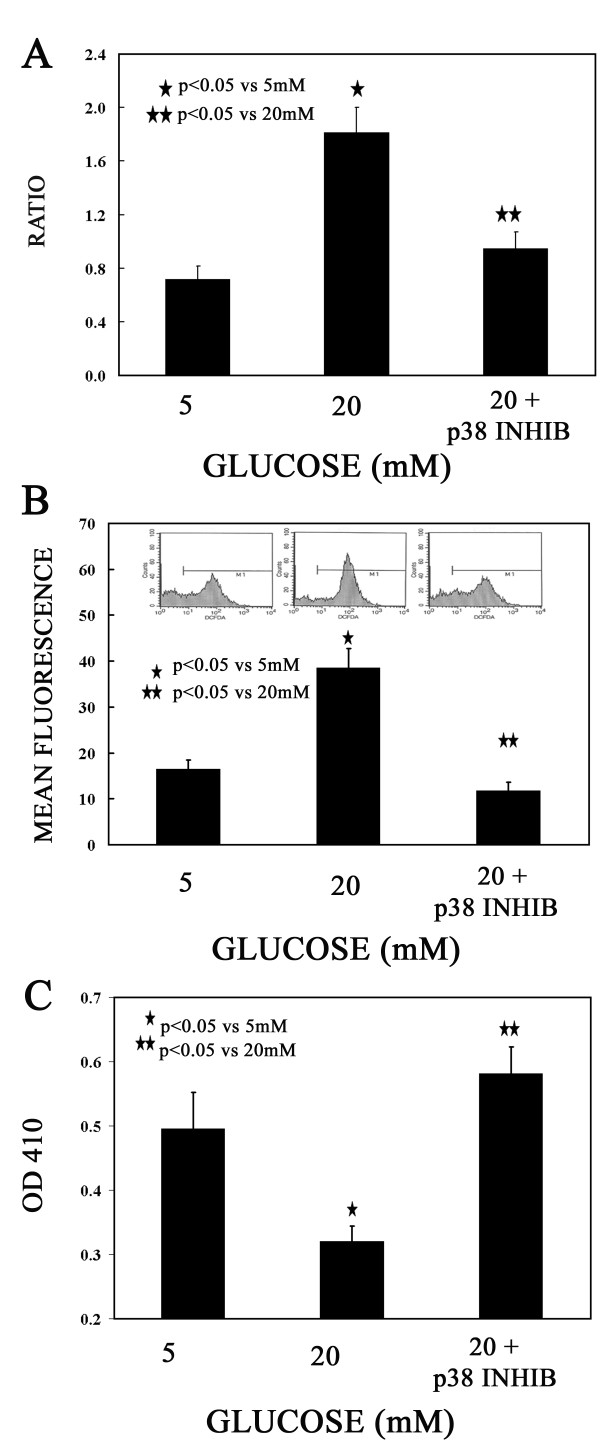
**ROS and TRX activity in response to glucose and p38 MAPK inhibition. **A) TXNIP RNA message levels were detected by semi-quantitative PCR in MDA-MB-231 cells grown either in 5 or 20 mM glucose chronically prior to plating. Average relative levels as compared to control β-actin RNA of TXNIP and TRX RNA messages from triplicates are represented. For inhibition of the p38 MAP kinase cells grown at 20 mM were pre-treated for 24 h with 20 μM SB203580. B) MDA-MB-231 cells were grown either in 5 or 20 mM glucose chronically prior to plating. Cells were assessed for ROS levels by DCFDA fluorescence staining and flow cytometry as shown in the inlet. For inhibition of the p38 MAP kinase cells grown at 20 mM glucose were treated for 24 h with 20 μM SB203580. Average mean fluorescence from triplicates is expressed per each group of cells. B) MDA-MB-231 cells were grown either in 5 or 20 mM glucose chronically prior to plating. Cells were assessed for TRX activity by the insulin disulfide reducing assay as described and the average OD 410 readings from triplicates are shown for each group of cells. For inhibition of the p38 MAP kinase cells grown at 20 mM were pre-treated for 24 h with 20 μM SB203580.

It has been recently shown that TXNIP binds to TRX and regulates the activity of this protein as a major cellular redox regulator [9,10]. Furthermore, hyperglycemia has also been shown to promote oxidative stress through inhibition of TRX function by TXNIP in human aortic smooth muscle cells (ASMCs) [12]. In order to assess whether hyperglycemia exerted any action in regulating ROS as an indicator of oxidative stress in response to 20 mM glucose (hyperglycemia)-induced elevation of TXNIP RNA level, we measured the ROS level as detected by CM-H2DCFDA and expressed as percentage of mean fluorescence by flow cytometry (Figure 4B, inlet) in relation to the level of TXNIP RNA. As shown in Figure 4A, as expected TXNIP RNA level expressed as ratio to control raised from baseline significantly (0.74 ± 0.07 at 5 mM glucose to 1.81 ± 0.14 at 20 mM glucose; p < 0.05). The increase of TXNIP RNA level was associated with 2.3 fold increase of ROS level in 20 mM vs 5 mM glucose (Figure 4B, 38.5% ± 4.2 for 20 mM vs 16.5% ± 2.0 for 5 mM glucose; p < 0.05). When we measured TRX activity, this decreased 1.6 fold from 5 to 20 mM glucose (0.496 ± 0.06 for 5 mM vs 0.320 ± 0.02 for 20 Mm glucose; p < 0.05) as shown in Figure 4C. Our data demonstrate that hyperglycemia-induced TXNIP elevation is associated with decreased TRX activity resulting in increasing levels of ROS in MDA-MB-231 cells.

### Hyperglycemia-induced TXNIP-ROS activity is mediated through p38 MAP kinase

A recent study has demonstrated that hyperglycemia-induced increased level of TXNIP is associated with activation of p38 MAP kinase in human ASMCs [12]. To assess whether p38 MAP kinase signaling pathway affected hyperglycemia-regulated TXNIP/TRX/ROS axis, we treated MDA-MB-231 cells with 20 μM of the specific kinase inhibitor SB203580. As illustrated in Figure 4A, TXNIP RNA level significantly dropped from 1.81 ± 0.14 to 0.94 ± 0.09 (p < 0.05) concordantly with the decreased ROS level (Figure 4B, from 38.5 ± 0.4 to 11.8 ± 0.2; p < 0.05). On the other hand, TRX activity significantly increased with the inhibitor (Figure 4C, from 0.320 ± 0.02 to 0.581 ± 0.04, p < 0.05). These data favor the functional relevance of p38 MAP kinase in metastatic breast-cancer derived cells MDA-MB-231 similarly to ASMCs as previously described [12].

## Discussion

In this study, we show that the metabolic condition of hyperglycemia affects the level of both TXNIP RNA and protein in breast-cancer derived cells MDA-MB-231. Thus the persistent elevation of TXNIP protein is strictly correlated with the persistency of the elevation of the glucose level and is very sensitive to its magnitude. In our cellular model, we have reproduced the conditions of postprandial hyperglycemia by shifting the glucose level from 5 to 20 mM and the conditions of insulin-resistant hyperglycemia (diabetes) by maintaining the cells "chronically" and stably at 20 mM glucose. We show that hyperglycemia by itself has a major impact on both the level of TXNIP and the regulation of ROS level/TRX activity in MDA-MB-231 cells. The regulation of TXNIP RNA level is related to the intracellular level of glucose and is notably reduced by the inhibition of the glucose transport. In fact, the increased uptake of glucose is relevant in regulating this function as demonstrated by the inhibition of the glucose transporter. Although phloretin did not completely inhibit the glucose-effect, the compound notably reduced TXNIP RNA. This observation may be explained by the presence of other GLUT proteins responsible for the glucose transport not inhibited by phloretin (*e.g*., compare 5 mM vs 20 mM in Figure 2B) as previously shown in breast cancer-derived cells [16]. However, the statistically significant reduction of TXNIP RNA obtained with phloretin-mediated inhibition of the major glucose transporter GLUT1 present in MDA-MB-231 cells supports the relevance of the intracellular glucose in the regulation of the TXNIP expression in MDA-MB-231 cells.

These data validate GEP reported by us and others that TXNIP represents a gene whose regulation is highly sensitive to glucose levels in metastatic breast cancer-derived MDA-MB-231 cells and in murine pancreatic β cells, respectively [1,7]. We also show that the regulation of the TXNIP/TRX/ROS axis is associated with p38 MAP kinase signaling pathway. These data are in agreement with previous observations in different cell models [12,17]. However, considering that MDA-MB-231 cells may have an increased baseline "hyperactive" MAP kinase activity due to ras-oncogene protein family dysregulation, the functional relevance of p38 MAP kinase in this and other breast cancer-derived cell lines needs to be further explored [18]. As shown herein, TXNIP-inhibiting TRX activity is highly regulated by glucose, hitherto we suggest that this protein may play a major role in translating the biological consequences of a metabolic condition such as diabetes in cancer biology. Recent studies have related the effect of hyperglycemia to increased generation of ROS and to greater DNA oxidative damage as main mechanism of accelerated aging and atherogenesis in the microangiopathic complications of the disease [12,17,19]. Although the relevance of diabetes in the pathogenesis and clinical course of tumors in general and particularly of breast cancer has been controversially debated, we illustrate for the first time in this context the molecular relationship between hyperglycemia and increased ROS production in a cancerous cellular model [2]. Our study opens new avenues for the investigation of the consequences of the described hyperglycemia-TXNIP-TRX-ROS axis on susceptibility to oxidative stress in oncogenesis and tumor progression. Although it has been proven that exogenous overexpression of TXNIP suppresses growth and induces apoptosis in vascular smooth muscle cells and cardiomyocytes, it has only been more recently that apoptosis and "glucotoxicity" have been related through TXNIP in murine pancreatic β cells [8,15]. However, in the current exploratory study, we did not address the ultimate effect of hyperglycemia-mediated TXNIP expression on apoptosis. Furthermore, it has been shown that glucose consumption rate is lower in non-invasive MCF-7 cells versus metastatic breast cancer-derived MDA-MB-231 cells used in the current study [20]. Gatenby et al. showed that up-regulation of glycolysis leads to microenvironmental acidosis as an evolutionary mechanism for the metastatic cell to adapt to the acidic microenvironment and promote proliferation and invasion [20]. Although the adaptive mechanism was well described, the question whether hyperglycemia represents a favorable or unfavorable condition for the cancerous cell that highly depends upon glycolysis was not posed [20]. Hitherto, future studies are warranted to assess whether hyperglycemia-induced TXNIP/TRX/ROS biology is a prerogative of MDA-MB-231 cells or it extends to other breast cancer-derived cell lines independently from their hormonal receptor status (estrogen receptors) or signaling pathways (ras mutations, p38 MAKP milieu, etc.). Although various studies have recently addressed the relevance of TXNIP in cancer biology, our study is the first one relating this protein to metabolic conditions of hyperglycemia/diabetes and oxidative stress in the same context [21-24]. Even though the incompleteness of this exploratory work that was meant to validate the preliminary findings of increased level of TXNIP observed in GEP in response to hyperglycemia hampers the understanding of the ultimate consequence of increased ROS for the growth of tumor cells in the metabolic conditions of hyperglycemia, we are confident that further investigative work will lead to the identification of the molecular mechanisms relating diabetes to cancer.

## Conclusion

Conclusively, we show that hyperglycemia finely regulates the expression of TXNIP in breast-derived cancer cells MDA-MB-231. The increased level of the protein leads to increased ROS levels through TRX reduced activity that is reversed by p38 MAP kinase inhibition. We describe an important signaling pathway that might be involved in diabetes-mediated oxidative stress in cancer. Our findings support further investigation in this perspective.

## List of abbreviations

Gene array profile (GPE); Thioredoxin-interacting protein (TXNIP); Vitamin D_3 _up-regulated protein-1 (VDUP-1); Insulin-like growth factors 1 (IGF-1); IGF binding proteins (IGFBPs); Sex hormone binding globulin (SHBG); Carbohydrate response elements (ChoRE); Thioredoxin (TRX); Reactive oxygen species (ROS); 5–6-chloromethyl-2',7'-dichlorodihydrofluorescein diacetate (CM-H2DCFDA); Glucose transporter 1 (GLUT1); aortic smooth muscle cells (ASMCs).

## Competing interests

The author(s) declare that they have no competing interests.

## Authors' contributions

FT has planned the project, designed the experimental plan and drafted the manuscript. EF has carried on the experimental plan with FT's assistance. TW has participated in the interpretation of the results and editing of the manuscript.

## Pre-publication history

The pre-publication history for this paper can be accessed here:


